# Effects of Dance on Pain in Patients with Fibromyalgia: A Systematic Review and Meta-Analysis

**DOI:** 10.1155/2018/8709748

**Published:** 2018-10-01

**Authors:** Álvaro Murillo-García, Santos Villafaina, José C. Adsuar, Narcis Gusi, Daniel Collado-Mateo

**Affiliations:** ^1^Faculty of Sport Science, University of Extremadura, Extremadura, Spain; ^2^Facultad de Educación, Universidad Autónoma de Chile, Talca, Chile

## Abstract

**Objective:**

The aim of this study was to perform a systematic review on the effectiveness of dance-based programs in patients with fibromyalgia, as well as calculate the overall effect size of the improvements, through a meta-analysis.

**Methods:**

The Cochrane Library, Physiotherapy Evidence Database (PEDro), PubMed, TRIP, and Web of Science (WOS) were selected to identify the articles included in this systematic review and meta-analysis. A total of seven articles fulfilled all inclusion and exclusion criteria. PRISMA guidelines were followed in the data extraction process. The level of evidence was established following guidelines from the Dutch Institute for Healthcare Improvement (CBO).

**Results:**

The studies were all randomized controlled trials, but not double-blind. Duration of dance programs ranged from 12 to 24 weeks. Sessions lasted between 60 and 120 minutes and were performed 1-2 times per week. The overall effect size for pain was -1.64 with a 95% CI from -2.69 to -0.59 which can be interpreted as large. In addition, significant improvements were observed in quality of life, depression, impact of the disease, anxiety, and physical function.

**Conclusion:**

Dance-based intervention programs can be an effective intervention for people suffering from fibromyalgia, leading to a significant reduction of the level of pain with an effect size that can be considered as large. However, findings and conclusions from this meta-analysis must be taken with caution due to the small number of articles and the large heterogeneity.

## 1. Introduction

Chronic pain conditions are responsible for the loss of quality of life and the increments in health care costs [1]. It also causes disability at work, generating periods of absence from work and early retirement [2]. The World Health Organization recognizes chronic pain as a public problem around the world. A systematic review showed the increasing prevalence of this condition, with 34% in low-income countries and 30% in high-income countries [3]. Fibromyalgia is one of the most common chronic pain diseases. It is characterized by widespread pain and stiffness, which persists for at least three months along with other associated symptoms such as sleep disturbance, poor physical fitness, cognitive impairment or anxiety, and depression [4]. In the European population (Spain, Portugal, France, Germany, and Italy) the estimated prevalence of fibromyalgia is between 2.9% and 4.7% [5].

Physical activity is a relevant therapy for the primary and secondary prevention of different chronic pain conditions [6], having specific benefits at reducing pain and increasing mental health and physical functions of people with chronic pain [7, 8]. Nonetheless, physical exercise is the nonpharmacological therapy with the highest level of evidence to reduce fibromyalgia symptoms [9]. Among physical exercise genres, dance has emerged as a relevant therapy to improve quality of life [10], cardiovascular mortality [11], or motivation to exercise [12] in special populations. Apart from the improvements associated with most types of physical exercise, dance comprises rhythmic motor coordination, cognitive, emotions, affection, and social interaction [13]. Besides, the artistic and creative components lead to further therapeutic benefits due to the integration of the bodily exterior with the psychic interior [14]. However, there are different types of dance that only involves the repetition of movements and lacks of creative, artistic, or emotional components. Thus, it could be possible that benefits from those types of dance are somewhat limited to the improvements of other physical exercise alternatives.

The artistic and creative concept of dance may be close to the notion of “creative arts therapies”, which are interventions that use artistic media to approach the participant on a creative level and may have benefits in different populations [15–17]. Through dance, patients can perform physical exercise but also develop the sense of self-control, which can lead to a decrease in feelings of anxiety that may contribute to the development of stress and pain experience [18].

There are numerous concretions of the social and cultural phenomenon of dance. Among those types of dance that fulfill with the previously mentioned concept of artistic and creative dance, we can mention the Dance Movement Therapy, Biodance, Aquatic Biodance, or Belly Dance. The American Dance Therapy Association (ADTA) defines Dance Movement Therapy as the psychotherapeutic use of movement to promote emotional, social, cognitive, and physical integration of the participant. Biodance involves the movement accompanied by music, inducing experiences capable of modifying the organism at the physiological, affective, motor, and existential levels [19]. Aquatic Biodance adds the benefits of water-based exercise programs [20, 21]. Belly Dance is an ancient form of dance which may promote physical rehabilitation, relaxation, social support, and body-mind connection [22].

To the best of our knowledge, there is no systematic review of the literature on the effectiveness of dance-based programs in patients with fibromyalgia [23]. Only one scoping review has been published in this area. However, in this review, authors did not report the effects of the included articles.

Therefore, the aim of this study was to systematically review the effectiveness of dance-based programs in patients with fibromyalgia, as well as calculate the overall effect size of the improvements through a meta-analysis.

## 2. Methods

The PRISMA (Preferred Reporting Items for Systematic reviews and Meta-Analyses) [24] guidelines have been followed to conduct the present systematic review.

### 2.1. Data Sources and Searches

**Figure 1:**
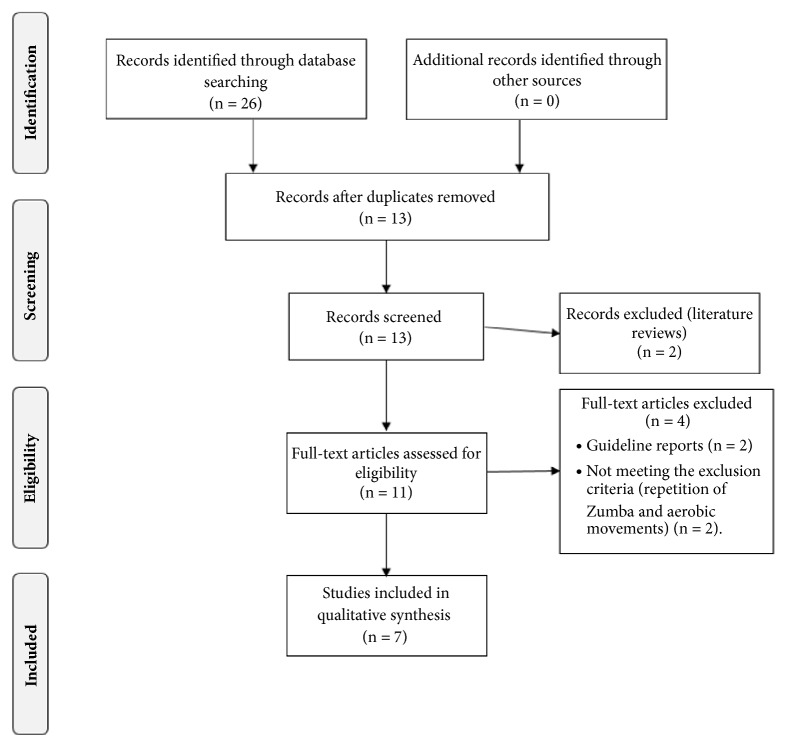
Study flow diagram.

The Cochrane Library, Physiotherapy Evidence Database (PEDro), PubMed, TRIP, and Web of Science (WOS) were selected to identify articles included in this systematic review. The search was performed using the keywords “fibromyalgia” and “dance” together with the logical operator “AND”. Search engines were configured to only present clinical trials. Duplicate articles were manually excluded.  Figure 1 shows the procedure performed for the selection of articles. These processes were developed by one author (AMG) and checked by another (JCA). The search was ended on June 20, 2018.

The articles were included if they met the following inclusion criteria: (a) the intervention program should be based on dance, (b) the target population were people with fibromyalgia, (c) the study was a randomized controlled trial (RCT), and (d) article was in English or Spanish. The following exclusion criteria were set: (a) the study was presented as a summary at a conference or seminar and (b) dance interventions involved only the repetitions of movements without a creative, emotional, or cultural components. This last criterion was previously used in a recent systematic review focused on the effectiveness of dance interventions to improve health in the elderly. That study defined dance as a form of artistic expression and excluded aerobic fitness classes taught to music, such as Zumba and step-aerobics [25]. Therefore, in the current systematic review, two studies [26, 27] were excluded because they were based on the repetition of Zumba movements.

### 2.2. Risk of Bias

To evaluate the risk of bias, the PEDro scale [28] was chosen due to its ability to provide an overall assessment of the external and internal validity of studies in the field of physiotherapy and health.  Table 1 shows the results of included articles.

### 2.3. Level of Evidence

The level of evidence was established using the guidelines from the Dutch Institute for Healthcare Improvement (CBO) [29]. The results of each of the articles are shown in Table 1.

### 2.4. Data Extraction

Data extraction was performed by one of the authors (AMG) and then checked by another author (JCA). The following information was extracted: participants, intervention, comparisons, results, and study design (PICOS) following the steps of the PRISMA methodology.  Table 2 summarizes the main characteristics of the sample, as well as the type of interventions performed by the control and dance groups. The details of the interventions, including durations of sessions and programs, the frequency, total time of interventions, and activities included in each session can be observed in Table 3. The effects of dance on pain can be seen in Table 4, while Table 5 summarizes the effects on other variables.

### 2.5. Statistical Analysis

A random effects model was employed to measure the effect of dance-based programs on pain. Table 4 shows the results of each study on this variable. The treatment effect was calculated as the difference between the change in the dance group and the change in the control group. Effect size was calculated with typical means and deviations before and after treatment [30]. For the meta-analysis, the magnitude of Cohen's d was defined as (a) small, for values between 0 and 0.5, (b) average, for values between 0.5 and 0.8, and (c) large, for values greater than 0.8. Heterogeneity was assessed by calculating the following statistics: (a) the Cochran Q-Test p value, (b) H, which is the square root of the Cochran Q-Test statistic divided by degrees of freedom and (c) I^2^, which is the transformation of the statistics used to determine the percentage of variation caused by heterogeneity. The most common I^2^ scale considers values lower than 25% as small heterogeneity, values between 25 and 50% as mean heterogeneity and values greater than 50% as great heterogeneity [31, 32]. All analyses were conducted using Microsoft Excel 2013 and the tool Meta-Easy [33].

## 3. Results

### 3.1. Article Selection

Figure 1 shows the complete process followed in this systematic review. A total of 26 articles were found in the electronic databases: the Cochrane Library (9 articles), PEDro (5 articles), PubMed (4 articles), TRIP (3 articles), and WOS (5 articles). Thirteen of those article were removed because they were duplicated and two because they were literature reviews. Of the remaining 13 articles, two were eliminated because they were guidelines and two because the intervention programs were not based on integrative dance (Zumba and aerobic dance). After this exhaustive selection, seven articles were included in the systematic review.

### 3.2. Risk of Bias

Table 1 shows the risk of bias of the articles, through the score obtained in the PEDro scale. The scores of the articles ranged from three to eight (the maximum score of the scale is 10). The mean of the scores was 5.27. The worst results were obtained in the items: 5 (“all subjects were blinded”) and 6 (“all the therapists who administered the therapy were blinded)”. The best results were obtained in the items: 1 (“the election criteria were specified”) that represents the external validity, 10 (“the results of statistical comparisons between groups were informed for at least one key result”), and 11 (“the study provides timely and variability measures for at least one key outcome”) that both represent statistical validity.

### 3.3. Level of Evidence

The level of evidence in each study is shown in Table 1. All the articles had a level of evidence B, because they are comparative studies but not double-blind.

### 3.4. Characteristics of the Study

Tables 2 and 3 show the characteristics of the studies following the stages of PICOS (P: participants; I: interventions; C: comparisons; O: outcomes; S: study design) established by the PRISMA methodology. The sample size of all the articles as a whole is 335 patients with fibromyalgia.

### 3.5. Interventions

Table 2 shows the different protocols of intervention in the dance groups of each article: two of Aquatic Biodance, two of Biodance, two of Dance Movement Therapy, and one of Belly Dance. This table also shows the protocols of the control groups in each article: four do not perform any alternative activity, two perform an exercise program based on stretching, and one performs a multidisciplinary exercise program.

Table 3 shows the durations and frequency of the dance-based interventions and a summary of session activities. Intervention durations ranged between 24 and 6 weeks. The weekly frequency of sessions is between one and two days, and the durations of these are between 60 and 120 minutes. The longest therapies were developed by Carbonell-Baeza A. et al. (2012) and Baptista A.S. et al. (2012), both with 32 total hours.

### 3.6. Measures and Effects on Pain

The effects of dance therapy on pain are shown in Table 4, reporting the change in each instrument. Five of the articles used a Visual Analog Scale (VAS), where three articles reported significant changes after the interventions [34–36]. This would mean a level of conclusion 2 according to the CBO [29].

In four of the articles, the Fibromyalgia Impact Questionnaire (FIQ) was used to measure pain. In three of them, significant changes were obtained [35–37].

In two of the studies the McGrill-Melzack Questionnaire was used to measure pain. In the study by López-Rodríguez, Castro-Sánchez, Fernández-Martínez, Matarán-Peñarrocha, and Rodríguez-Ferrer [35] intragroup significant reductions were observed in the dance group while López-Rodríguez, Fernández-Martínez, Matarán-Peñarrocha, Rodríguez-Ferrer, Gámez, and Ferrándiz [36] reported between groups significant differences.

In addition, in four of the articles the algometer was used. In two of them there were significant changes [36, 37]. In the study of López-Rodríguez, Castro-Sánchez, Fernández-Martínez, Matarán-Peñarrocha, and Rodríguez-Ferrer [35] the total score of the algometer is not reported, with the comparison with other articles being impossible.

In two of the articles the SF-36 was used to measure pain. In one of them, authors reported significant changes [34].

Bojner-Horwitz, Kowalski, Theorell, and Anderberg [38] also reported significant changes in the Global Assessment of Well-Being and Pain.

### 3.7. Measures and Effects on Other Variables

The effects of dance therapy on other variables are shown in Table 5, reporting the change in each instrument.

Quality of life was measured in three articles through the SF-36. In this regard, two of the articles reported changes in the social dimension [37, 39]. However, in the study by Carbonell-Baeza, Ruiz, Aparicio, Martins-Pereira, Gatto-Cardia, Martinez, Ortega, and Delgado-Fernandez [39], the differences were in favor of the control group. The third article [34] reported changes in emotional and mental health dimensions. In five articles the sleep quality was measured, three presented significant changes, two were measured with the FIQ [35, 37], and one was measured with the Pittsburgh Sleep Quality Index [36].

There are eleven measures of the depression symptoms but these measures were obtained with different instruments. In this regard, the most used tool was the FIQ (four articles), the Beck Inventory (two articles), and the Hospital Anxiety and Depression Scale (two articles). However, significant changes were only observed in two articles, [35, 37], measured through the FIQ.

Regarding anxiety, three studies reported significant changes [35–37], using the FIQ and the Anxiety State Inventory.

The impact of the disease was assessed in five article using the FIQ [34–37, 39], obtaining significant changes. In the study of [39] there were only within group differences.

Cardiorespiratory functional capacity was assessed in three articles through the “6-minute walk test”. Two articles reported significant improvements [34, 37]. Moreover, two articles reported agility tests results (“8-Feet Up and Go” test), with only one achieving significant changes in the dance group [37]. Regarding mobility, [40] reported significant improvements in the 5-Point Video Interpretation Scale.

In addition, two articles reported significant differences in self-image, using the Examination of Body Dysmorphic Disorder [34] and the Autofigure Drawing Procedure [38].

### 3.8. Meta-Analysis

**Figure 2:**
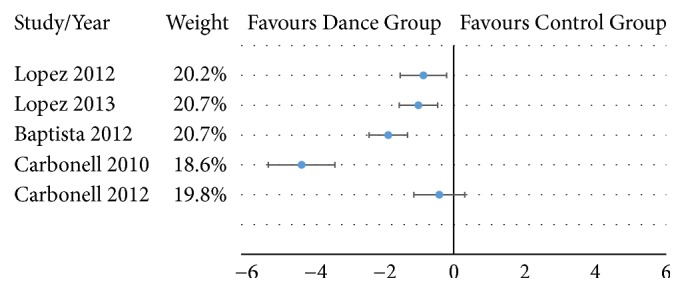
Effect sizes for pain of the articles included in the meta-analysis.

In Figure 2, which represents the meta-analysis, only 5 articles are included [34–37, 39]. Two articles were not included because they did not report enough data to calculate effect sizes [38, 40].

The overall effect size for pain was -1.64 with a 95% CI from -2.69 to -0.59 which is large according to the proposed classification (>0.8). The p-value was lower than 0.01 which means a significant reduction in pain. The heterogeneity level was large as the p-value of the Cochran Q-test was <0.01. Additionally, this was supported by the other computed coefficients: I^2^=92% and H=3.57. Publication bias statistics are not reported because analyses based on the funnel plot are not recommended when there are less than 10 articles and the heterogeneity is large [41, 42].

## 4. Discussion

This systematic review and meta-analysis aims to explore the effectiveness of dance-based intervention programs for patients with fibromyalgia. The main finding was that dance significantly reduces the level of pain in people with fibromyalgia. This reduction was observed in all five studies and can be considered as large according to the overall effect size (effect size of -1.64 with a 95% CI from -2.69 to -0.59 and p-value <0.01). Given the relevance of pain symptoms in fibromyalgia syndrome, there is evidence that therapies based on dance may be beneficial and clinically relevant for people with fibromyalgia. However, the large heterogeneity, the low number of studies, and the different types of control groups make that those conclusions must be taken with caution.

It must be noted that there were relevant differences in the interventions. Although all included articles were based on dance and have common aspects, there are differences due to the conception of the modality. In order to reduce the heterogeneity of dance interventions and following the criteria previously used by Hwang and Braun [25], the current systematic review and meta-analysis only included those articles with interventions based on the artistic expression and excluded those studies aimed at evaluating the effects of Zumba or step-aerobics.

The different interventions based on dance included in this review were Biodance, on both land [37, 39] and aquatic [35, 36], Dance Movement Therapy (DMT) [38, 40], and Belly Dance [34]. The highest effect size was obtained after the on-land biodance intervention is carried out by Carbonell-Baeza et al. [37]. However, the lowest effect size that can be seen in Figure 2 was found with the same protocol [39]. Although this could seem contradictory, the control group in the last study carried out a multidisciplinary program that included 48 sessions of aquatic and land-based exercises and psychological therapy. This multidisciplinary program reduced pain from 7.9 to 6.3, while biodance improve pain was reduced from 8.2 to 6.4. Therefore, the effect size in that study is affected by the benefits of the multidisciplinary program. On the other hand, the study with the highest effect size is influenced by the increment of pain levels in the control group (from 7.3 to 8.0). Nonetheless, all 5 articles included in the meta-analysis reported higher reduction of pain in the dance group compared with the control group.

Regarding aquatic biodance, there were other relevant benefits apart from pain. Participants improved their fatigue, stiffness, anxiety, depression, and the impact of the disease. This may be in line with previous studies reporting that physical exercise in warm water is recommended for the management of chronic pain due to warm temperature and the buoyancy [43–46]. Therefore, the combination of dance and aquatic exercise may lead to higher improvements due to the well-known properties of physical exercise in warm water and the potential benefits of the artistic expression in dance programs.

Depression symptoms were significantly reduced in three of the seven studies [35–37]. According to a previous research [47], the minimal clinically important difference on the Beck Depression Inventory was 17.5%, which is higher than the changes observed after any of the interventions that used that questionnaire. To our knowledge, the minimal clinically important difference has not been reported for the rest of the depression instruments used. These results are in line with a previous review [48], concluding that there is low-quality evidence regarding the effectiveness of Dance Movement Therapy as an intervention to reduce depression. Interestingly, only those three articles observed significant differences in anxiety. This reduction may be related to the artistic expression of creative arts therapies [15] and the potential effects of listening to music in blood pressure, heart rate, respiratory rate, or anxiety [49]. The current systematic review does not provide enough information to draw solid conclusions about the effects of dance interventions on anxiety and depression symptoms in people with fibromyalgia; thus further studies are needed.

Although only artistic and creative dance interventions were included, other studies have investigated the effect of rhythmic exercises like Zumba on different variables in people with fibromyalgia. However, the poor methodological quality and the low number of participants make the extraction of conclusions very hard. The study by Assunção Júnior, de Almeida Silva, da Silva, da Silva Cruz, de Almeida Lins, and de Souza [27] observed improvements in pain and physical function, but the design only included one group; thus comparisons are not possible. Also, a recent study evaluated the effectiveness of a virtual reality intervention that included one environment focused on Zumba [50, 51]. Authors clearly stated that dance steps were marked by a professional kinesiologist and dance teacher, with no artistic nor creative component.

Impact of fibromyalgia was also reduced after dance interventions. All articles that used the FIQ observed a reduction that was higher than 14%, which is considered the minimal clinically important difference in fibromyalgia patients. This finding, along with the improvements in pain supports the notion of the benefits of dance in this population. The growing interest on new technologies and virtual reality may complement the dance-based interventions. Previous studies have showed an improvement in pain, quality of life, mobility skills, balance, and fear of falling, supporting the notion that physical exercise performed through virtual reality may be an excellent alternative to improve chronic pain [50, 51]. Therefore, future studies may explore the potential of virtual reality to conduct dance-based interventions focused on the reduction of pain in fibromyalgia patients.

The current systematic review and meta-analysis has some limitations. First, search was limited to studies in Spanish and English indexed in the mentioned electronic databases. Although all journals indexed in the Journal of Citations Reports are indexed in the consulted databases, it could be possible that some articles written in other languages have been omitted. Second, two of the studies did not report enough data to be included in the meta-analysis; thus only five of the seven articles were included, which means a relatively small sample size. Finally, findings and conclusions from this systematic review and meta-analysis must be taken with caution due to the large heterogeneity observed, the possible publication bias, and the different types of control groups.

## 5. Conclusions

Dance-based intervention programs can be an effective intervention for people suffering from fibromyalgia, leading to a significant reduction of the level of pain with an effect size that can be considered as large. Also, dance may improve quality of life and physical function, as well as reducing anxiety, depression, and the impact of the disease. However, findings and conclusions from this meta-analysis must be taken with caution due to the small number of articles and the large heterogeneity.

## Figures and Tables

**Table 1:** Risk of bias and level of evidence.

**Validity**	External	Internal								Statistics		PEDro Score	Level of Evidence
**Criterion**	1	2	3	4	5	6	7	8	9	10	11		

**Bojner-Horwitz, E. et al 2003**	✓	✓								✓	✓	3	B
**Bojner – Horwitz, E. et al 2006**	✓	✓	✓	✓			✓	✓	✓	✓	✓	8	B
**Carbonell-Baeza, A. et al 2010**	✓			✓				✓	✓	✓	✓	5	B
**Lopez- Rodriguez, M.M. et al 2012**	✓			✓				✓	✓	✓	✓	5	B
**Carbonell-Baeza, A. el al 2012**	✓			✓				✓	✓	✓	✓	5	B
**Baptista, A.S. et al 2012**	✓	✓	✓	✓			✓	✓	✓	✓	✓	8	B
**Lopez- Rodriguez, M.M. et al 2013**	✓	✓		✓			✓	✓	✓	✓	✓	7	B

✓: criteria fulfilled; 1: the election criteria were specified; 2: the subjects were randomly assigned to the groups (in a cross-over study, the subjects were randomly distributed as they received the treatments); 3: the assignment was hidden; 4: the groups were similar at the beginning in relation to the most important prognostic indicator; 5: all subjects were blinded; 6: all the therapists who administered the therapy were blinded; 7: all evaluators who measured at least one key result were blinded; 8: the measurements of at least one of the key results were obtained from more than 85% of the subjects initially assigned to the groups; 9: results were presented for all subjects who received treatment or were assigned to the control group, or when this could not be, the data for at least one key result were analyzed by “intention to treat”; 10: the results of statistical comparisons between groups were informed for at least one key result; 11: the study provides punctual and variability measures for at least one key outcome; score: each criterion met (except the first) adds 1 point; B: level of evidence from randomized studies but without double blindness,

**Table 2:** Characteristics of the sample and the protocol.

	Characteristics of the Sample		Protocol	
**Study/Year**	Size (N)	Age (SD)	Control Group Treatment	Dance Based Group Treatment
**Bojner-Horwitz, E. et al 2003**	36^∗^	57 (7.20)	None	Dance Movement Therapy

**Bojner – Horwitz, E. et al 2006**	36^∗^	57 (7.20)	None	Dance Movement Therapy

**Carbonell-Baeza, A. et all 2010**	59^∗^ 71^∗∗^	54.2 (6.2) EG 51.4 (7.4) CG	None	Biodance

**Lopez- Rodriguez, M.M. et al 2012**	39^∗^ 70^∗∗^	55.41 (7.46)	Stretching exercise program: global and specific stretches of different muscle areas.	Biodance in a pool with a water temperature of 29°C, preceded by a shower at 33-35°C.

**Carbonell-Baeza, A. el al 2012**	31^∗^ 38^∗∗^	54.5 (7.5) EG 50.9± 7.7 CG	Multidisciplinary program based on: Pool exercises. Activity in the exercise room. Psychological-Educational Therapy.	Biodance

**Baptista, A.S. et al 2012**	75^∗^ 80^∗∗^	49.5 EG 49.1 CG	None	Belly Dance

**Lopez- Rodriguez, M.M. et al 2013**	59^∗^ 76^∗∗^	55.45 (7.80) EG 54.23 (7.21) CG	Stretching exercise program: Slow and coordinated breathing stretches.	Biodance in a pool with a water temperature of 29°C, preceded by a shower at 33-35°C.

*∗*: n for the analysis of the effect of the treatment; *∗∗*: n for the analysis of the intention to treat effect; EG: experimental group; CG: control group; N/A: not applicable; SD: standard deviation.

**Table 3:** Duration and activities of the intervention.

**Study/Year**	Intervention duration (weeks)	Session duration (minutes)	Weekly frequency (days)	Total time (hours)	Activities included in session
**Bojner-Horwitz, E. et al 2003**	24	60	1	24	Exercises based on different themes: Body, space and group awareness. Expression and quality of movement. Movement about feelings, images and words. Differentiation of feelings and integration. Video interpretation.

**Bojner – Horwitz, E. et al 2006**	24	60	1	24	Exercises based on different themes: Body, space and group awareness. Expression and quality of movement. Movement about feelings, images and words. Differentiation of feelings and integration.

**Carbonell-Baeza, A. et al 2010**	12	120	1	24	35-45 minutes, verbalization phase (information and experiences). 75-80 minutes, phase of dance movements according to the demands of the instructor and music, individually, in pairs and in groups.

**Lopez- Rodriguez, M.M. et al 2012**	12	60	2	24	10 minutes, warm-up and stretching. 40 minutes, biodance movements (walking or slow movements of limbs). 10 minutes, stretching.

**Carbonell-Baeza, A. el al 2012**	16	120	1	32	35-45 minutes, verbalization phase (information and experiences). 75-80 minutes, phase of dance movements according to the demands of the instructor and music, individually, in pairs and in groups.

**Baptista, A.S. et al 2012**	16	60	2	32	Warm up exercises, movements of upper limbs, scapular area, trunk, hip, displacements (progression in difficulty). Exercises back to calm. CD at home to practice exercises.

**Lopez- Rodriguez, M.M. et al 2013**	12	60	2	24	10 minutes, flexibility and breathing. 40 minutes, basic movements of expression and creative dance including upper and lower members, according to music, expressing emotions generated by melodies, other people and interaction. All with cooperation between members. 10 minutes, relaxing exercises.

**Table 4:** Effects of dance interventions on pain.

**Study/Year**	Instrument	Measurement range	Sample		Activity		CG				EG				P	Effect on CG	Effect on EG	Found Effect	Effect Size
			n CG	n EG	CG	EG	Mean Pre-T	SD	Mean Post-T	SD	Mean Pre-T	SD	Mean Post-T	SD					
**Bojner-Horwit, E. et al 2003**	VAS	0-10	16	20	None	DMT	NR	NR	NR	NR	NR	NR	NR	NR	D				

**Bojner-Horwit, E. et al 2006**	VAS	0-10	16	20	None	DMT	NR	NR	NR	NR	NR	NR	NR	NR	D				
	Global Assessment of Well-being and Pain	0-5	16	20	None	DMT	NR	NR	NR	NR	NR	NR	NR	NR	A				

**Carbonell-Baeza, A. et al 2010**	Algometer EFFEGI, FPK 20, Italy)	0-90	32	27	None	Biodance	50.3	1.77	47.29	1.91	48.38	1.94	53.39	2.08	A	-3.01	5.01	8.02	4.33
	Algometer EFFEGI, FPK 20, Italy)	0-18	32	27	None	Biodance	16.16	0.38	16.38	0.46	16.77	0.42	15.32	0.5	A	0.22	-1.45	-1.67	-4.18
	FIQ	0-10	32	27	None	Biodance	7.3	0.3	8	0.3	6.9	0.4	6.1	0.3	AB	0.7	-0.8	-1.5	-4.29

**Lopez- Rodriguez, M.M. et al 2012**	VAS	0-10	20	19	Stretch.	Aquatic Biodance	8.3	0.73	7.95	1.19	7.16	2.34	5.42	2.19	B	-0.35	-1.74	-1.39	-0.22
	Questionnaire McGill - Melzack	0-78	20	19	Stretch.	Aquatic Biodance	39.7	8.31	35.25	4.88	39.58	3.3	28.68	6.69	B	-4.45	-10.9	-6.45	-0.22
	Algometer Wagner FPI 10 – USA		20	19	Stretch.	Aquatic Biodance													
	FIQ	0-10	20	19	Stretch.	Aquatic Biodance	7.07	1.8	6.69	1.75	7.2	1.68	5.33	2.12	A	-0.38	-1.87	-1.49	-0.28

**Carbonell-Baeza, A. et al 2012**	Algometer (FPK 20; Effegi, Alfonsine, Italy)	0-90	18	13	Multid.	Biodance	44.55	2.3	50.26	2.76	48.8	3.09	56.05	3.71	D	5.71	7.25	1.54	0.57
	Algometer (FPK 20; Effegi, Alfonsine, Italy)	0-18	18	13	Multid.	Biodance	17.02	0.43	15.83	0.82	16.36	0.58	13.8	1.1	D	-1.19	-2.56	-1.37	-2.75
	SF- 36	0-100	18	13	Multid.	Biodance	18.6	3	32.7	4.8	23.7	3.8	30	6.1	D	14.1	6.3	-7.8	-2.32
	FIQ	0-10	18	13	Multid.	Biodance	7.9	0.4	6.3	0.4	8.2	0.6	6.4	0.5	D	-1.6	-1.8	-0.2	-0.40

**Baptista, A.S. et al 2012**	VAS	0-10	37	38	None	Belly Dance	7.5	1.3	7.3	1.7	7.7	1.7	4.7	2.6	A	-0.2	-3	-2.8	-1.84
	SF-36	0-100	37	38	None	Belly Dance	25.7	13.4	29.1	21.1	29.6	17.5	46	19.2	A	3.4	16.4	13	0.83

**Lopez-Rodriguez, M.M. et al 2013**	VAS	0-10	30	29	Stretch.	Aquatic Biodance	7.77	1.1	7.33	1.3	7.17	1.8	5.17	2.1	AB	-0.44	-2	-1.56	-0.27
	Questionnaire McGill - Melzack	0-78	30	29	Stretch.	Aquatic Biodance	39.43	8.2	37.2	6.2	39.28	3.3	29.97	6.7	AB	-2.23	-9.31	-7.08	-0.25
	Algometer Wagner FPI 10 – USA	0-18	30	29	Stretch.	Aquatic Biodance	14.7	3.4	13.63	2.6	14.72	3.9	9.66	4.2	AB	-1.07	-5.06	-3.99	-0.36
	FIQ	0-10	30	29	Stretch.	Aquatic Biodance	7.7	1.2	7.8	1.1	6.93	1.9	5.45	1.8	AB	0.1	-1.48	-1.58	-0.27

EG: experimental group; CG: control group; n: sample size; SD: standard deviation T: test; P: P-value; VAS: visual analog scale; FIQ: fibromyalgia impact questionnaire; NR: not reported; N/A: not applicable; A: significant changes of experimental group versus control group; B: changes in experimental group; C: changes in group control; D: no significant changes; Multid.: multidisciplinary; Stretch.: stretching; DMT: Dance Movement Therapy.

**Table 5:** Effects on other variables.

**Study/Year**	Instrument	Measures	CG				EG				P.	Found Effect
			Mean Pre-T	SD	Mean Post-T	SD	Mean Pre-T	SD	Mean Post-T	SD		
**Bojner-Horwitz, E. et al 2003**	Blood Samples	Dehydroepiandrosteronsulphate	NR	NR	NR	NR	NR	NR	NR	NR	D	
	Blood Samples	Cortisol	NR	NR	NR	NR	NR	NR	NR	NR	D	
	Blood Samples	Prolactin	NR	NR	NR	NR	NR	NR	NR	NR	D	
	Blood Samples	Neuropeptide Y	NR	NR	NR	NR	NR	NR	NR	NR	D	
	Saliva Samples	Cortisol	NR	NR	NR	NR	NR	NR	NR	NR	D	
	Montgomery Asberg Rating Scale	Depression	NR	NR	NR	NR	NR	NR	NR	NR	D	
	Video-interpretation Rating Scale	Mobility	NR	NR	NR	NR	NR	NR	NR	NR	A	
	Video-interpretation Rating Scale	Perception of Movement Pain	NR	NR	NR	NR	NR	NR	NR	NR	A	
	Video-interpretation Rating Scale	Life Energy	NR	NR	NR	NR	NR	NR	NR	NR	A	

**Bojner – Horwitz, E. et al 2006**	Self-Figure Drawing Procedure	Self-Image										
		Amount of Body Details	11.9	1.9	8.9	1.9	11.1	1.9	16.1	1.9	A	8
		Amount of paper use in percent	50	7.4	40	6.2	48.8	7.4	68.8	6.2	A	30
		Amount of Kilo Bytes when scanning	136.8	6.9	135.5	12.1	148.4	6.9	156	12.1	D	8.9
		Amount of Colours	3.5	0.6	3.3	0.6	3.3	0.6	3.8	0.6	D	0.7
	Comprehensive Psychopathologic Rating Scale	Depression	NR	NR	NR	NR	NR	NR	NR	NR	D	
	Sense of Coherence	Coping Capacity of Life-Stressors	NR	NR	NR	NR	NR	NR	NR	NR	D	
	Swedish Universities Scale of Personality	Personality Profile	NR	NR	NR	NR	NR	NR	NR	NR	D	
	Global Assessment of Well-Being and Pain	Well-Being	NR	NR	NR	NR	NR	NR	NR	NR	A	

**Carbonell-Baeza, A. et al 2010**	Eight-Polar Tactile-Electrode Impedanciometer (InBody 720, Biospace)	Body Composition										
		Weight in Kilograms	68.5	2.1	68.8	2	68.1	2.2	67.5	2.2	D	-0.9
		Waist Circumference	87.8	1.9	86.1	1.9	87.1	1.9	86.5	1.9	D	1.1
		Body Mass Index	28.2	0.9	28.3	0.9	27.5	0.9	27.4	0.9	D	-0.2
		Body Fat Percentage	38.6	1.2	37.2	1.6	37.2	1.2	31.4	1.6	AB	-4.4
		Muscle Mass in Kilograms	22.6	0.5	22.7	1.4	23.3	0.5	27.2	1.5	A	3.8
	“30-Second Shair Stand” Test	Lower Body Muscular Strength	7	0.5	8	0.5	8	0.5	10	0.5	B	1
	Digital Dynamometer	Upper Body Muscular Strength	15.7	1	17.3	1	18.1	1	18.4	1.1	D	-1.3
	“Chair Sit and Reach” Test	Lower Body Flexibility	-13.2	2.7	-15.7	2.9	-11	2.8	-6.3	3	D	7.2
	“Back Scratch” Test	Upper Body Flexibility	-7.3	2.4	-9.3	2.4	-6.5	2.4	-5.8	2.5	D	2.7
	“Blind Flamingo” Test	Static Balance	10	1	11	1	10	1	9	1	D	-2
	“8-Feet Up and Go” Test	Motor Agility	8.3	0.3	7.8	0.3	7.6	0.3	6.8	0.3	B	-0.3
	“6-Minute Walk” Test	Aerobic Endurance	456.6	12.7	457	13.1	448.7	13.5	480.9	13.8	A	31.8
	FIQ	Impact of the Syndrome										0
		Physical Function	4.3	0.3	4.8	0.4	4.4	0.4	3.6	0.4	A	-1.3
		Fell-good	8.3	0.4	8.8	0.4	7.6	0.4	6.1	0.5	AB	-2
		Fatigue	8.2	0.3	8.5	0.3	7.8	0.4	6.5	0.3	AB	-1.6
		Morning Tiredness	8	0.3	8.11	0.4	8.4	0.3	6.4	0.4	A	-2.11
		Stiffness	7.6	0.4	7.9	0.4	6.6	0.4	6	0.5	B	-0.9
		Anxiety	7.4	0.4	7.9	0.4	6.2	0.5	5.2	0.5	AB	-1.5
		Depression	6.1	0.5	7	0.5	5.7	0.6	4.9	0.6	A	-1.7
		Final Score	70.1	2.1	74	2.8	66.9	2.9	56	3.1	AB	-14.8
	SF-36	Health-related quality of Life										
		Physical Functioning	39.1	3.5	38	3	38.1	3.8	44.8	3.2	D	7.8
		Physical Role	5.2	3.3	3.3	2.6	6.8	3.6	10	2.8	D	5.1
		General Health	26.5	3	29	3.1	33	3.2	35.6	3.4	D	0.1
		Vitality	18.1	2.8	19	2.9	22.6	3	26.4	3.2	D	2.9
		Social Functioning	44.4	4.4	36.7	3.7	49.2	4.8	55.6	4	AB	14.1
		Emotional Role	33.4	8	38	8.1	39.4	8.8	48.8	8.9	D	4.8
		Mental Health	45.4	3.6	44.9	4.2	50.8	3.9	57.9	4.6	D	7.6
	Hospital Anxiety and Depression Scale	Anxiety	11.2	0.8	11	0.8	9.4	0.9	9.1	0.9	D	-0.1
		Depression	9.3	0.7	9	0.8	7.5	0.8	7.3	0.9	D	0.1
	Vanderbilt Pain Management Inventory	Coping Strategies										
		Passive Coping	24.7	0.8	24.2	0.7	23.2	0.9	20.7	0.7	D	-2
		Active Coping	16.1	0.7	16.1	0.7	16.5	0.7	16	0.7	D	-0.5
	Rosenberg Self-Esteem Scale	Self-Esteem	28.2	1.1	25.4	1.2	28.4	1.2	28.3	1.3	A	2.7
	General Self-Efficacy Scale	Self-Efficacy	25	1.3	25.5	1.3	26.9	1.4	27.9	1.4	D	0.5

**López- Rodriguez, M.M. et al 2012**	FIQ	Impact of the Syndrome										
		Daily Life Activities	1.27	0.45	0.99	0.44	1.17	0.58	0.62	0.57	A	-0.27
		Number of Days That Was Good the Last Week	0.75	1.11	0.85	1.26	1.05	1.12	2.21	2.01	A	1.06
		Absent Days at Work	0.92	1.55	0.84	1.67	1.1	2.07	0.55	1.66	D	-0.47
		Fatigue	7.85	1.18	7.45	1.39	7.95	1.35	6.31	1.85	A	-1.24
		Morning Tiredness	8.15	1.03	7.55	1.66	7.95	1.26	6.89	1.85	D	-0.46
		Stiffness	7.6	1.46	7.55	1.57	6.95	1.98	6.15	2	A	-0.75
		Anxiety	7.15	1.26	7.9	1.37	7.16	1.6	5.52	2.26	A	-2.39
		Depression	6.65	2	8.3	1.17	5.74	2.53	4.42	2.41	A	-2.97
		Inconvenience on Work	7.8	1.28	7.2	1.57	7.58	1.53	5.42	2.19	A	-1.56
		Final Score	69.55	12.96	69.23	12.89	67.08	10.51	52.16	16.18	AB	-14.6
	Beck Inventory	Depression	19.35	7.7	16.7	6.66	18.05	9.83	16.05	7.39	D	0.65

**Carbonell-Baeza, A. el al 2012**	Eight-Polar Tactile-Electrode Impedanciometer (InBody 720, Biospace)	Body Composition										
		Weight in Kilograms	68.3	2.4	68.3	2.5	69	3.1	68	3.3	D	-1
		Waist Circumference	86.9	2.7	88.1	2.8	87.2	3.4	85.5	3.5	D	-2.9
		Body Mass Index	27.9	1.1	28	1.2	27.8	1.4	27.8	1.5	D	-0.1
		Body Fat Percentage	38.4	1.7	37.8	1.9	36.5	1.9	35.9	2.2	D	0
		Muscle Mass in Kilograms	22.3	0.7	27.4	3.3	23.9	0.8	22.6	3.9	D	-6.4
	“30-Second Chair Stand” Test	Lower Body Muscular Strength	8	0.6	8.2	0.6	7.7	0.8	8.2	0.7	D	0.3
	“Chair Sit and Reach” Test	Lower Body Flexibility	-17.6	4.5	-7.7	2.9	-15.7	5.8	-6.9	3.7	D	-1.1
	“Back Scratch” Test	Upper Body Flexibility	-7.3	2.4	-9.3	2.4	-6.5	2.4	-5.8	2.5	D	2.7
	“8-Feet Up and Go” Test	Motor Agility	8.1	0.4	7.9	0.4	7.8	0.5	6.6	0.5	D	-1
	“6-Minute Walk” Test	Aerobic Endurance	449.6	16.3	445.8	14.6	443.9	20.3	461	18.1	D	20.9
	Dinamómetro Digital	Upper Body Muscular Strength	14.8	1.6	16.1	1.3	17.9	1.9	20.4	1.6	D	1.2
	“Blind Flamingo” Test	Static Balance	12.2	1.1	10.6	1.3	9.6	1.5	9.6	1.7	D	1.6
	FIQ	Impact of the Syndrome										
		Physical Function	5.4	0.5	4	0.5	4.4	0.6	4.3	0.7	D	1.3
		Well-Being	8.7	0.5	7.8	0.7	8.6	0.6	6.7	0.8	D	-1
		Fatigue	8.5	0.4	7.6	0.5	8.4	0.6	7.7	0.6	D	0.2
		Morning Tiredness	8.3	0.4	8.1	0.4	8.9	0.6	8.3	0.6	D	-0.4
		Stiffness	7.6	0.5	6.1	0.6	8.3	0.7	7	0.8	D	0.2
		Anxiety	8.5	0.4	5.9	0.6	7.7	0.5	6.9	0.8	D	1.8
		Depression	7	0.7	4.9	0.7	7.8	0.9	6.3	0.9	D	0.6
		Final Score	74.6	3.1	62.9	3.7	77.7	3.9	64.9	4.7	BC	-1.1
	SF-36	Health-related quality of Life										
		Physical Functioning	35.8	4.7	42.4	4.8	35	6	38.3	6.2	D	-3.3
		Physical Role	0	0	9.7	4.6	9.1	5.6	4.5	5.9	A	-14.3
		General Health	27.7	3.2	31.9	3.3	26	4	31.9	4.2	D	1.7
		Vitality	20.4	4	23.5	3.5	16.2	2.1	25.2	4.5	D	5.9
		Social Functioning	31.7	6.1	51.6	5.8	44.3	7.8	43.9	7.5	A	-20.3
		Emotional Role	25.5	8.5	42.7	10.3	15.8	10.8	12	13.2	D	-21
		Mental Health	41.7	4.5	53.3	4.6	37.3	5.8	44	5.9	D	-4.9
	Hospital Anxiety and Depression Scale	Anxiety	11.9	1	10.4	1	12.1	1.3	11.9	1.3	D	1.3
		Depression	9.6	1.1	8.5	1	9.1	1.4	8.5	1.3	D	0.5
	Vanderbilt Pain Management Inventory	Coping Strategies										
		Passive Coping	25.6	0.7	21.4	0.9	23.7	0.9	22.2	1.2	A	2.7
		Active Coping	15.6	0.9	17	0.9	15.9	1.1	14.2	1.1	D	-3.1
	Rosenberg Self-Esteem Scale	Self-Esteem	29.1	1.4	29.2	1.3	26.4	1.2	27.1	1.7	D	0.6
	General Self-Efficacy Scale	Self-Efficacy	26.1	1.9	27.1	1.5	24.1	2.4	23.1	1.9	D	-2

**Baptista, A.S. et al 2012**	“6-Minute Walk” Test	Functional Capacity	332	66.7	343	77.9	372.8	80.2	431	88.7	A	47.2
	FIQ	Impact of the Syndrome	6.34	1.29	5.9	1.86	5.89	1.39	4.26	1.81	A	-1.19
	SF-36	Health-related quality of Life										
		Physical Functioning	32.6	18.9	39.1	22	44.9	1.89	56.3	19.9	D	4.9
		Physical Role	8.8	17.9	13.8	26.5	24.7	32.2	36.5	32.4	D	6.8
		General Health	38	16.5	41.5	21.4	46	21.7	44.9	15.6	D	-4.6
		Vitality	29	18.2	37.1	21.8	41.3	18.8	47.6	23.8	D	-1.8
		Social Functioning	47.6	23.1	51.3	25.5	52.6	27.7	57.2	27	D	0.9
		Emotional Role	21.2	33.1	31.5	38.7	34.2	36.9	51.9	39.6	A	7.4
		Mental Health	43.4	24	46.2	22.6	46	19.9	52.3	20.8	A	3.5
	Beck Inventory	Depression	21.2	13	23.5	13.7	23.9	14.7	23.1	15.3	D	-3.1
	State-Trait Anxiety Inventory, Part 1	Anxiety	52.8	8.1	51.8	9.4	50.5	7.68	49.4	10	D	-0.1
	State-Trait Anxiety Inventory, Part 2	Anxiety	53	9.7	54.1	10.1	51.1	7.8	49.8	9.1	D	-2.4
	Body Dysmorphic Disorder Examination	Self-Image	48.8	34.2	46.9	31.8	42.8	32.6	41.1	24.24	A	0.2

**López- Rodriguez, M.M. et al 2013**	FIQ	Impact of the Syndrome										
		Daily Life Activities	1.28	0.4	1.4	0.5	1.12	0.5	0.65	0.6	AB	-0.59
		Number of Days That Was Good the Last Week	0.9	1.2	1	1.3	1	1.1	2.07	2.1	AB	0.97
		Absent Days at Work	1.33	1.6	1.22	1.8	0.73	1.7	0.38	1.3	D	-0.24
		Fatigue	6.89	1.6	6.56	1.5	6.87	1.8	4.46	2.4	AB	-2.08
		Morning Tiredness	7.77	1.3	7.57	1.2	7.59	1.3	6.66	1.9	D	-0.73
		Stiffness	7.8	1.1	7.53	1.5	7.59	1.3	7.17	2	D	-0.15
		Anxiety	7.03	1.5	6.47	1.7	6.86	1.8	6.41	1.9	D	0.11
		Depression	7.1	1.3	6.97	1.2	7.03	1.5	5.9	2.5	AB	-1
		Inconvenience on Work	6.53	1.9	6.4	1.7	5.66	2.5	4.52	2.4	D	-1.01
		Final Score	68.63	14.1	68.5	14.1	65.79	11.2	53.73	18	AB	-11.95
	Pittsburgh Sleep Quality Index	Sleep Quality	14.7	2.4	14.4	2.4	15.1	1.7	7.59	1.8	AB	-7.21
	Center for Epidemiologic Studies Depression Scale	Depression	26.37	6.4	27.7	7.3	23.07	9.7	20.41	11.1	D	-3.99
	State Anxiety Inventory	Anxiety	44.97	4.5	44.87	5.1	45.17	3.9	38.79	5.8	AB	-6.28

EG: experimental group; CG: control group; n: sample size; SD: standard deviation; T: test; FIQ: Fibromyalgia Impact Questionnaire; NR: not reported; NA: not applicable; A: significant changes of experimental group versus control group; B: changes in experimental group; C: changes in control group; D: not significative changes; P: P-value.
